# Protective Effects of Emodin on Oxidized Fish Oil-Induced Metabolic Disorder and Oxidative Stress through Notch-Nrf2 Crosstalk in the Liver of Teleost *Megalobrama amblycephala*

**DOI:** 10.3390/antiox11061179

**Published:** 2022-06-15

**Authors:** Changyou Song, Bo Liu, Hongxia Li, Yongkai Tang, Xianping Ge, Bo Liu, Pao Xu

**Affiliations:** 1 Key Laboratory of Aquatic Animal Nutrition and Health, Freshwater Fisheries Research Center, Chinese Academy of Fishery Science, Wuxi 214081, China; songchangyou@ffrc.cn (C.S.); lihx@ffrc.cn (H.L.); tangyk@ffrc.cn (Y.T.); gexp@ffrc.cn (X.G.); 2 Wuxi Fisheries College, Nanjing Agricultural University, Wuxi 214081, China; liubo@njau.edu.cn

**Keywords:** oxidative stress, Notch-Nrf2 crosstalk, antioxidant, metabolism, emodin, *Megalobrama amblycephala*

## Abstract

Dietary oxidized lipids are key perpetrator to accumulate excessive reactive oxygen species (ROS) that induce oxidative stress for animals. Immoderate oxidative stress dysregulates cell fate, perturbs cellular homeostasis, thereby interrupts metabolism and normal growth. Therefore, a 12-week feeding trial with fish oil (FO, control group), oxidized fish oil (OF), and emodin-supplemented (OF+E) diets was conducted to evaluate the therapeutic mechanism of emodin on metabolic and oxidative resistance in *Megalobrama amblycephala* liver. Morphologically, emodin remits oxidized fish oil-induced cellular constituents damage, evidenced by lipid droplets enlargement and accumulation, mitochondria rupture, and nucleus aggregation, which were functionally related to oxidative stress, metabolism, and cell fate determination. Consecutively, glucose, lipid, and amino acid metabolism were retained under emodin stimulation. Specifically, fatty acid metabolic genes optimized fatty acid utilization and metabolism, featured as total saturated fatty acids (SFA), monounsaturated fatty acids (MUFA), and polyunsaturated fatty acids (PUFA) alternation. Physiologically, inflammation, autophagy, apoptosis, as well as antioxidant capacity were alleviated by emodin. Interactively, fatty acid metabolism was correlated with antioxidant capacity; while the crosstalk and dynamic equilibrium between apoptosis and autophagy determine the cell fate under oxidative stress amelioration. Synergistically, Nrf2 and Notch signaling were active to antioxidant defense. In particular, oxidative stress blocked the crosstalk between Notch and Nrf2 signaling, while emodin rescued Notch-Nrf2 interaction to ameliorate oxidative stress. In conclusion, these results suggest that elevated ROS levels by oxidative stress activates Notch and Nrf2 signaling but intercepts Notch-Nrf2 crosstalk to stimulate cell fate and antioxidant program; dietary emodin alleviates oxidative stress and returns overall ROS levels to a moderate state to maintain homeostatic balance. The crosstalk between Notch and Nrf2 signaling might be the potential therapeutic target for emodin to ameliorate oxidative stress and metabolic disorder in *M. amblycephala* liver.

## 1. Introduction

Internal homeostasis is an important precondition for animal physiological health, growth, and reproduction maintenance. Reactive oxygen species (ROS) and reactive nitrogen species (RNS) are generated during cellular metabolism, which play important roles in internal homeostasis, cell signal transduction, cell proliferation and differentiation [1]. Generally, animals are exposed to internal or external adverse environments that could dysregulate ROS or RNS homeostasis, which trigger oxidative stress consequently. Oxidative stress is the most extensive and harmful stress among all stresses. When oxidative stress exceeds the repair capacity of the cell, oxidative damage will lead to physiological disorder, lipid peroxidation, DNA alteration, protein degradation, immunity and growth inhibition, and even severe death [2].

Adequate energy intake and nutritional balance are important prerequisites for aquatic animal growth and reproduction. However, excessive or insufficient nutrient intake, as well as nutrient deterioration, causes an imbalance between pro-oxidants and antioxidants, which was coined as “dietary oxidative stress” [3]. Dietary oxidative stress could induce metabolic disorder and homeostasis imbalance, leading to oxidative stress and inflammatory responses [4], disrupt cellular homeostasis and cell fate turnover, thereby seriously affecting the health of aquatic animals and causing severe loss to the aquaculture industry [5]. Fat is an important nutrient and energy substance, which can provide essential fatty acids for fish, promote the absorption and utilization of fat-soluble nutrients such as vitamins, as well as maintain the stability of biofilm structure [6]. In aquaculture, fish oil and soybean oil are the most commonly used animal- and plant-based lipids. However, due to the high content of unsaturated fatty acids, they are easily to be oxidized under natural conditions. Therefore, oxidative stress caused by nutritional imbalance, especially by dietary oxidized lipids that accompanies the entire life cycle of aquatic animals, should be paid enough attention. In view of this, considering lipid oxidation-induced oxidative stress to study oxidative stress in aquatic animals, clarifying the molecular mechanism and targets of oxidative stress injury, and exploring effective stress prevention strategies are both representative and of great significance to the health development of aquaculture.

It is clear that deleterious effects of ROS are controlled by specific antioxidant systems [7]. Two potential mechanisms may be involved in increase of ROS generation by oxidized fish oil: one is the conjugation of products of metabolism with glutathione, resulting in consumption of GSH and decreasing the defense potential leading to oxidative stress [8]. Another one is the antioxidant defense system to reduce the oxidative damage, including antioxidant enzymes and signaling molecules [8]. Nowadays, attentions have been attracted to deciphering regulatory pathways in which free radicals are involved. To date, many pathways have been investigated in detail, including Nrf2/Keap1 and Notch signaling.

Nrf2 (nuclear factor, erythroid derived 2, like 2) is a nuclear transcription factor that is critical for cellular protection and cell survival [9]. Notch signaling regulates cell fate decision in animals, such as cell differentiation, survival, apoptosis, and cell cycle in both physiologic and pathologic contexts [1]. The role of Nrf2 signaling in ROS-induced oxidative stress has been well studied, and recent reports also reveal ROS could activate Notch pathway [10]. As the major effector of ROS, Nrf2 regulates a number of ARE-containing genes, including Notch1, to regulate cell fate and reduce ROS levels in the cell [10]. Meanwhile, it has been clearly shown that NICD can activate the Nrf2 pathway and Nrf2 can activate the Notch1 pathway in the liver [11]. Additionally, Notch-Nrf2 signaling was active to promote self-renewal of the liver and intestine cell by autophagy and apoptosis under ROS-induced oxidative stress [12,13]. Therefore, the crosstalk between Notch and Nrf2 is critical for liver metabolism and cell fate determination.

In recent years, dietary intake has become the main approach for stress control and prevention in aquatic animals, including probiotics, prebiotics, microbial feed additives, and natural functional feed additives [14]. These functional feed additives could improve antioxidant performance and reduce oxidative stress by chelating metal ions, regulating intestinal microorganisms, increasing antioxidant factor activity, and inhibiting ROS over-production [14]. However, the underlying molecular mechanism is still not fully investigated, which has become an urgent scientific bottleneck for oxidative stress prevention and control in aquatic animals.

Emodin is a natural anthraquinone derivative that is enriched in many widely used Chinese medicinal herbs, such as *Rheum palmatum*, *Polygonum cuspidatum*, and *Polygonum multiflorum* [15]. Emerging evidence indicates that emodin possesses a wide spectrum of pharmacological properties, including anticancer, hepatoprotective, anti-inflammatory, antioxidant, and antimicrobial activities [16]. Previously, we elucidate dietary emodin ameliorates dietary oxidized fish oil-induced oxidative stress by improving antioxidant and immune capacity, reducing intestinal cell autophagy, and thereby rescuing the growth performance of *M. amblycephala* [17]. In particular, PPARs, Nrf2, and Notch signaling were involved in the regulation [1,17,18]. Apart from the intestine, liver is constantly exposed to environmental oxidants and therefore serves as an interesting model system to study oxidative stress. However, the regulation on metabolism and antioxidant, and the underlying mechanism between Notch and Nrf2 signaling in the liver still remains unclear. 

In the present study, we investigated the systematic alternation among metabolism, immunity, and antioxidant capacity under oxidative stress in the liver of *M. amblycephala*. Meanwhile, the role of Notch and Nrf2 signaling, as well as their crosstalk in resist to oxidative stress and emodin amelioration were also identified. These results could reveal the mechanism and will provide potential therapeutic targets for emodin in aquaculture.

## 2. Materials and Methods

### 2.1. Ethics Statement

This study was approved by the Animal Care and Use Committee of Nanjing Agricultural University (Nanjing, China; protocol code: WXFC 2017-0006, approved 27 May 2017). All animal procedures were carried out in accordance with the China Laboratory Animal Care and Use Guidelines. The ethics in this experiment is the same as the ethics previously published [18]. 

### 2.2. Experimental Diets

According to our previous research, oxidized fish oil in the diet has been shown to induce oxidative stress [18], and emodin was confirmed to improve the antioxidant capacity in *M. amblycephala* [19]. Therefore, this experiment applied oxidized fish oil with a peroxide value (POV) of 375.33 mmol/kg to induce oxidative stress with the fish model of *M. amblycephala*. Specifically, isonitrogen and isoenergy (33.11% crude protein and 14.68 kJ/g energy) diets were formulated, including basal diets containing 6% fish oil (control, 6F), oxidized lipid enriched diets containing 6% oxidized fish oil (6OF), and emodin enriched diets containing 30 mg/kg emodin (6OF+E) (Table 1). All ingredients were prepared and the diets were pelleted according to the established methods [19]. 

### 2.3. Experimental Animals and Rearing Conditions

The experimental fish *M. amblycephala* was generated from our research institute Freshwater Fisheries Research Center, Chinese Academy of Fishery Sciences. The experiment was conducted in fiberglass tanks (300 L each) of indoor fresh water circulation system to support equal supplemental aeration and water flow (3 L/min). Prior the feeding trail, fish were acclimated in the tank fed with the control feed for 14 days. After acclimation, fish with similar body weight (intimal average weight 5.20 ± 0.01 g) were randomly assigned to nine tanks (3 tanks for each group, 25 individuals for each tank, 225 fish in total). During the 12-week rearing experiment, fish were fed with the respective diets to near satiation four times a day (8:00, 11:00, 14:00, and 17:00). During the experiment, one-third of the water in the tank was replaced weekly, the water temperature was kept as 26 ± 1 °C, and the water quality maintained as: pH 7.6–7.8, DO > 6 mg/L, NH_3_ < 0.01 mg/L.

### 2.4. Sample Collection

After a 12-week feeding experiment, the fish were starved for 24 h to evacuate the digestive tract contents before sampling. Nine fish in each group were taken (three fish from each tank were randomly selected) and anesthetized with tricaine mesylate (MS-222, 100 mg/L) for sampling. Blood samples were obtained from caudal vein and stored in heparin coated tubes, and then centrifuged at 4500 rpm, 4 °C for 10 min to obtain the plasma. The anesthetized fish were then dissected to remove the liver tissues on ice, immediately isolated for TEM analysis or frozen in liquid nitrogen and stored at −80 °C for subsequent analysis.

### 2.5. Liver Histological Ultrastructure

According to our previously established method [18], TEM was used to detect histological ultrastructure. In detail, the livers of *M. amblycephala* (3 replicates per group) were collected immediately and fixed in 2.5% glutaraldehyde for 24 h, post-fixed in 1% osmium tetroxide (OsO4) for 1 h, and stored at 4 °C till sectioning. The sections were embedded in epoxy resin Epon812, cut into thin slices (70 μm thick) with RMC PowerTome XL microtome, and stained with uranyl acetate and lead citrate. A Hitachi HT7700 transmission electron microscope (Hitachi, Tokyo, Japan) was used to observe the ultrastructure morphology.

### 2.6. Metabolic and Antioxidant Index Detection

Glucose content in the plasma, digestive enzyme activity of amylase, alkaline phosphatase (ALP), lipase, lipoprotein lipase (LPL), triglyceride (TG), total cholesterol (TC), and fatty acid synthetase (FAS) in the liver was determined by assay kits according to the manufacturer’s protocol (provided by Nanjing Jiancheng Bioengineering Institute, Nanjing, China). In detail, glucose was detected by hexokinase method (Category No: F006-1-1), amylase was determined by starch-iodine colorimetry method (Category No: C016-1-1), ALP was determined by colorimetric method (Category No: A059-2), lipase was determined by colorimetric method (Category No: A054-1-1), LPL was determined by colorimetric method (Category No: A067-1), TG was determined by colorimetric method (Category No: A110-2), TC was determined by colorimetric method (Category No: A111-2), and FAS was determined by (Category No: A080-2-2).

Meanwhile, antioxidant capacity-related index of reactive oxygen species (ROS), total superoxide dismutase (T-SOD), inducible nitric oxide synthase (iNOS), nitric oxide (NO), reduced glutathione (GSH), glutathione peroxidase (GPx), anti-superoxide anion (ASAFR) and malondialdehyde (MDA) were all determined by assay kits according to the manufacturer’s protocol (provided by Nanjing Jiancheng Bioengineering Institute, China). The category numbers of the kit were as following: ROS, E004-1; T-SOD, A001-1; iNOS, H372-1; NO, A012-1; GSH, A006-2; GPx, A005-1; ASAFR, A052-1; and MDA, A003-1.

### 2.7. Fatty Acid and Amino Acid Analysis

For fatty acid analysis, whole fish samples were hydrolyzed with BHT for 1.5 h, neutralized with ddH_2_O and n-hexane, and centrifuged at 1000 rpm for 10 min. The supernatant was isolated to analyze fatty acid composition by meteorological chromatography-mass spectrometer (GC-MS, Aglilent 7890B-5977A).

For amino acid analysis, whole fish samples were hydrolyzed with 6 mol/L HCl and filled with nitrogen for 24 h. The samples were applied to determine the amino acid content by liquid chromatography analyzer (Agilent-1100).

### 2.8. Correlation Analysis

Pearson’s correlation test was performed to analyze the correlations between parameters or key genes. The significance threshold was set at a *p*-value < 0.05. The heatmap was created in R with the pheatmap package.

### 2.9. RNA Extraction and RT-PCR Analysis

Total RNA from nine livers in each group was extracted with RNAiso Plus reagent (Takara Co. Ltd., Dalian, China), and the total RNA was incubated with RNase-free DNase (Takara Co. Ltd., Dalian, China) to remove contaminated genomic DNA. Absorbance under OD 260/280 and electrophoresis (1.5% agarose) was applied to evaluate the quantity and quality of RNAs. Primers for RT-PCR was designed with primer Premier 5.0 according to the sequence we obtained with RNA-seq and synthesized by Shanghai Generay Biotechnology Co., Ltd., China (primers were shown in Table 2). RT-PCR analysis was performed using SYBR^®^ Primix Ex TaqTM II (TliRNase Plus) kit according to the manufacturer’s protocol with ABI 7500 real-time PCR system. β-actin was used as the housekeeping gene, and the relative expression was calculated using the 2^−ΔΔCT^ method.

### 2.10. Statistical Analysis

To determine the variances for each parameter among different groups, data were all validated for normality and homogeneity, followed by independent samples *t*-test with SPSS 25.0 (IBM, Chicago, IL, USA). Results were expressed as mean ± standard error of the mean (mean ± SEM).

## 3. Results

### 3.1. Emodin Alleviates Oxidized Fish Oil Induced Morphological Impairment in the Liver of M. amblycephala

**Figure 1:**
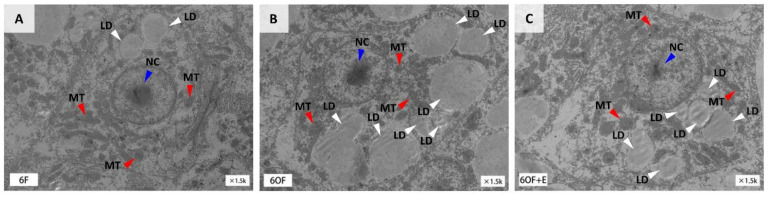
Emodin alleviates oxidized fish oil-induced morphological impairment in the liver of *M. amblycephala.* (**A**–**C**), Ultra-structure of the liver obtained from transmission electron microscopy (TEM), (**A**), 6% fish oil (6F); (**B**), 6% oxidized fish oil (6OF); (**C**), 6% oxidized fish oil with 30 mg/kg emodin (6OF+E). Arrows in white color represent the lipid droplets (LD), in red represent the mitochondria (MT), and in blue represent the nucleolus (NC), *n* = 3.

TEM was applied to reveal the histological alternation of the liver tissue induced by dietary oxidized fish oil and emodin. In comparison with the control group (6F, Figure 1A), lipid droplets (LD) were enlarged in size and accumulated in quantity (Figure 1B, arrows in white color), mitochondria (MT) were ruptured gradually (Figure 1B, arrows in red color), and nucleolus (NC) was aggregated (Figure 1B, arrows in blue color) under oxidized fish oil (6OF). These morphological impairments indicate 6OF-induced oxidative stress, metabolic disorder, and cell fate dysregulation in the liver. Heartily, we found the structure of LD, MT, and NC was visibly rescued under emodin stimulation (6OF+E), indicating the ameliorative effects of emodin on oxidative stress, cellular metabolism, and cell fate determination (Figure 1C).

### 3.2. Emodin Alleviates Metabolic Disorder Induced by Oxidative Stress in the Liver of M. amblycephala

**Figure 2:**
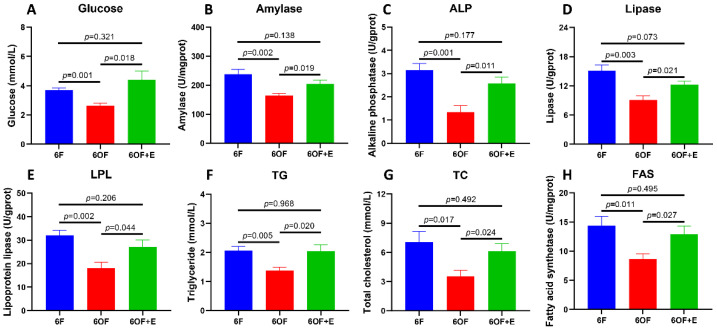
Emodin alleviates metabolic disorder induced by oxidative stress in the liver of *M. amblycephala.* (**A**–**G**) represent the glucose, lipid, and protein metabolic related indexes detected from the liver. (**A**), glucose; (**B**), amylase; (**C**), alkaline phosphatase (ALP); (**D**), lipase; (**E**), lipoprotein lipase (LPL); (**F**), triglyceride (TG); (**G**), total cholesterol (TC); (**H**), fatty acid synthetase (FAS). Data were analyzed by Students’ *t*-test, results were indicated as mean ± SEM, *n* = 9.

To reveal the metabolic alterations retrieved form the mitochondria histology, glucose, lipid, and protein metabolic-related indexes were detected with the liver tissue. Glucometabolic-related glucose content (Figure 2A) and amylase activity (Figure 2B) were inhibited by 6OF; protein metabolic-related alkaline phosphatase (ALP) activity (Figure 2C) was also inhibited by 6OF; lipid and fatty acid metabolic related lipase (Figure 2D), lipoprotein lipase (Figure 2E), triglyceride (Figure 2F), total cholesterol (TC, Figure 2G), and fatty acid synthetase (Figure 2H) were all inhibited by 6OF. However, the aberrant expression of these genes was rescued by emodin (6OF+E), and exhibited no significant difference with that in 6F (*p* ≥ 0.05) (Figure 2A–H).

### 3.3. Emodin Rescues Fatty Acid Metabolism under Oxidative Stress in the Liver of M. amblycephala

**Figure 3:**
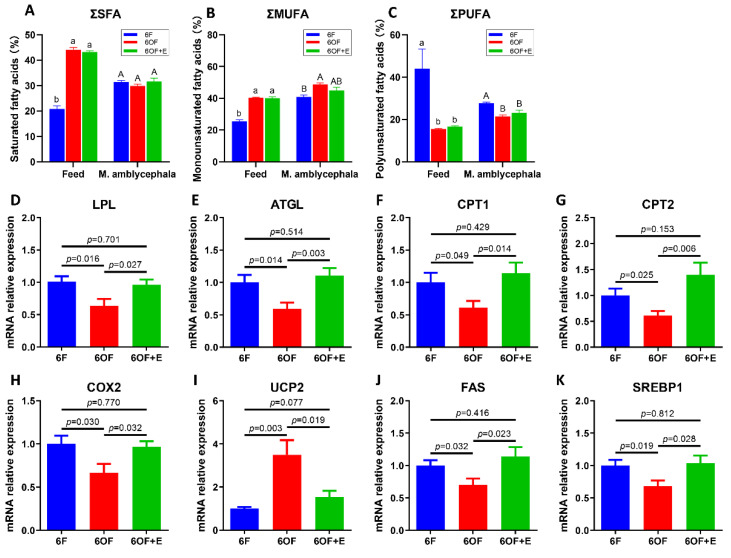
Emodin rescues fatty acid metabolism under oxidative stress in the liver of M. amblycephala. (**A**–**C**), fatty acid composition in the feed and M. amblycephala. (**A**), total saturated fatty acids (∑SFA); (**B**), total monounsaturated fatty acids (∑MUFA); (**C**), total polyunsaturated fatty acids (∑PUFA). (**D**–**I**), transcriptional expression of fatty acid catabolic related genes. (**D**), Lipoprotein lipase (LPL); (**E**), adipose triglyceride lipase (ATGL); (**F**), carnitine palmitoyltransferase I (CPT1); (**G**), carnitine palmitoyltransferase II (CPT2); (**H**), cyclooxygenase 2 (COX2); (**I**), uncoupling protein 2 (UCP2). (**J**,**K**), transcriptional expression of fatty acid anabolic related genes. (**J**), Fatty acid synthase (FAS); (**K**), sterol regulatory element-binding protein 1 (SREBP1). In panel (**A**–**C**), letters “a and b” represent the difference in the feed, “A and B” represent the difference in the M. amblycephala. Data were analyzed by Students’ *t*-test, results were indicated as mean ± SEM, *n* = 9.

To further explore the potential alteration of fatty acid metabolism that is regulated by OF, fatty acid composition, fatty acid catabolism, and anabolism were analyzed. Fatty acid composition of the diet reveal saturated fatty acids (SFA) and monounsaturated fatty acids (MUFA) were significantly increased (*p* < 0.05), while the polyunsaturated fatty acids (PUFA) were significantly decreased (*p* < 0.05) after the oxidization of fish oil (Figure 3A–C, Table 3). However, the fatty acid composition from the whole fish reveal SFA exhibited no significant difference between 6F, 6OF, and 6OF+E (*p* > 0.05); MUFA in 6OF was increased, but restored to the 6F level under emodin (6OF+E); PUFA in 6OF and 6OF+E was significantly decreased (*p* < 0.05), and exhibited the same variation as that in the diet (Figure 3A–C, Table 3). These results indicate *M. amblycephala* presents positive adaptability to fatty acid metabolism under fatty acid composition alteration in the diet induced by oxidized fish oil, especially the SFA and MUFA utilization. This interesting finding inspired us to explore the fatty acid catabolic and anabolic regulation.

In the fatty acid catabolism process, we found the transcriptional expression of lipoprotein lipase (LPL, Figure 3D), adipose triglyceride lipase (ATGL, Figure 3E), carnitine palmitoyltransferase I (CPT1, Figure 3F), carnitine palmitoyltransferase II (CPT2, Figure 3G), and cyclooxygenase 2 (COX2, Figure 3H) were all inhibited by 6OF; while uncoupling protein 2 (UCP2, Figure 3I) was activated by 6OF. Meanwhile, in fatty acid anabolic process, transcriptional expression of fatty acid synthase (FAS, Figure 3J) and sterol regulatory element-binding protein 1 (SREBP1, Figure 3K) were also down-regulated under 6OF. However, results also show the expression of these key genes were restored to the control level under 6OF+E stimulation (Figure 3D–K).

### 3.4. Emodin Alleviates Antioxidant Capacity under Oxidative Stress in the Liver of M. amblycephala

**Figure 4:**
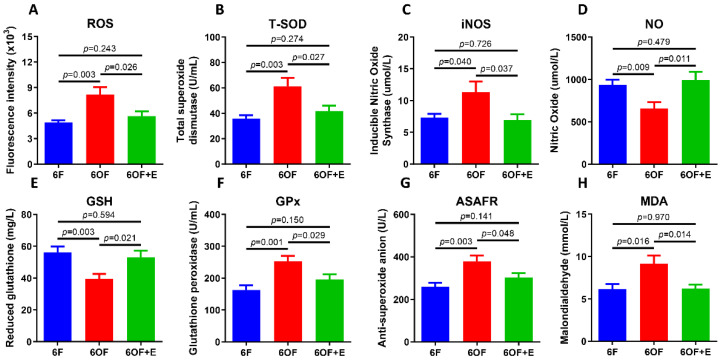
Emodin alleviates antioxidant disorder under oxidative stress in the liver of *M. amblycephala.* (**A***–***G**), Antioxidant related indexes. (**A**), Reactive oxygen species (ROS); (**B**), total superoxide dismutase (T-SOD); (**C**), inducible nitric oxide synthase (iNOS); (**D**), nitric oxide (NO); (**E**), reduced glutathione (GSH); (**F**), glutathione peroxidase (GPx); (**G**), anti-superoxide anion (ASAFR); (**H**), malondialdehyde (MDA). Data were analyzed by Students’ *t*-test, results were indicated as mean ± SEM, *n* = 9.

To evaluate whether dysregulated metabolism impacts antioxidant capacity, we next evaluated the antioxidant-related parameters in the liver. 6OF significantly increased the content or activity of reactive oxygen species (ROS, Figure 4A), total superoxide dismutase (T-SOD, Figure 4B), inducible nitric oxide synthase (iNOS, Figure 4C), glutathione peroxidase (GPx, Figure 4D), anti-superoxide anion (ASAFR, Figure 4E), and malondialdehyde (MDA, Figure 4F); and significantly decreased the content of nitric oxide (NO, Figure 4G) and reduced glutathione (GSH, Figure 4H). Analogously, emodin (6OF+E) also alleviates the increased or decreased activity or content to the control level after 12-weeks stimulation (*p* > 0.05, Figure 4A–H).

To reveal the relationship between fatty acid metabolism and antioxidant capacity, a correlation analysis was conducted with Pearson analysis (Figure S1). Under oxidative stress (6OF), fatty acid metabolism-related indexes were synergistically correlated with antioxidant enzyme activities, but the correlation indexes were different with that in 6F. However, after emodin stimulation (6OF+E), the synergy was reduced with the only correlation of LPL and NO, indicating the regulation between fatty acid metabolism and antioxidant capacity were closely related to emodin stimulation.

### 3.5. Emodin Alleviates Inflammation, Autophagy and Apoptosis under Oxidative Stress

**Figure 5:**
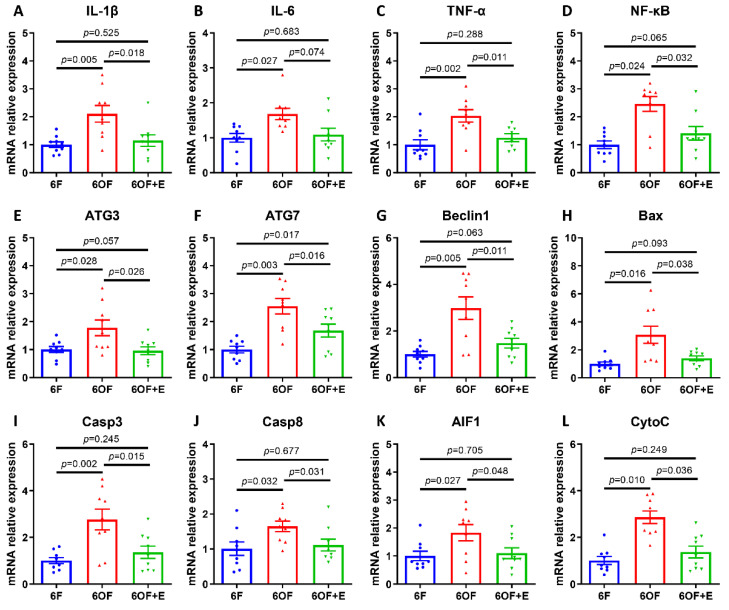
Emodin alleviates inflammation, autophagy, and apoptosis under oxidative stress in the liver of *M. amblycephala.* Transcriptional expression of inflammation-related (**A***–***D**), autophagy-related (**E***–***G**), and apoptosis-related (**H***–***L**) genes. (**A**), Interleukin 1 beta, (IL-1β); (**B**), interleukin 6 (IL-6); (**C**), tumor necrosis factor alpha (TNF-α); (**D**), nuclear factor kappa B (NF-κB); (**E**), autophagy-related 3 (ATG3); (**F**), autophagy-related 7 (ATG7); (**G**), Beclin1; (**H**), BCL2-Associated X (Bax); (**I**), caspase 3 (Casp3); (**J**), caspase 8 (Casp8); (**K**), apoptosis inducing factor 1 (AIF1); (**L**), cytochrome complex (CytoC). Data were analyzed by Students’ *t*-test, results were indicated as mean ± SEM, *n* = 9.

To uncover whether the metabolic and physiological alternation was related to immunity and cell fate determination, transcriptional expression of inflammation, autophagy, and apoptosis-related key genes were detected. Consistently, 6OF activated inflammatory response (IL-1β, IL-6, TNF-α, and NF-κB, Figure 5A–D), cellular autophagy (ATG3, ATG7, and Beclin1, Figure 5E–G), and apoptosis (Bax, Casp3, Casp8, AIF1, and CytoC, Figure 5H–L). Simultaneously, emodin (6OF+E) alleviates the expression of these genes (Figure 5A–L), and reveals emodin exerts protective effects on inflammation and cellular homeostasis under oxidative stress. Moreover, correlation analysis (shown in Figure S2) reveal inflammation factor IL-1β was associated with apoptosis factor Bax and AIF1 in 6OF; inflammation and autophagy correlation were vanished in 6OF and 6OF+E; while apoptosis and autophagy correlation were activated in 6OF (Casp9-ATG7) and 6OF+E (Casp8-ATG3, Casp8-ATG7). These data indicate the crosstalk and dynamic equilibrium between apoptosis and autophagy determines the cell fate under oxidative stress amelioration.

### 3.6. Notch-Nrf2 Crosstalk Was Active to Oxidative Stress Amelioration in the Liver of M. amblycephala

**Figure 6:**
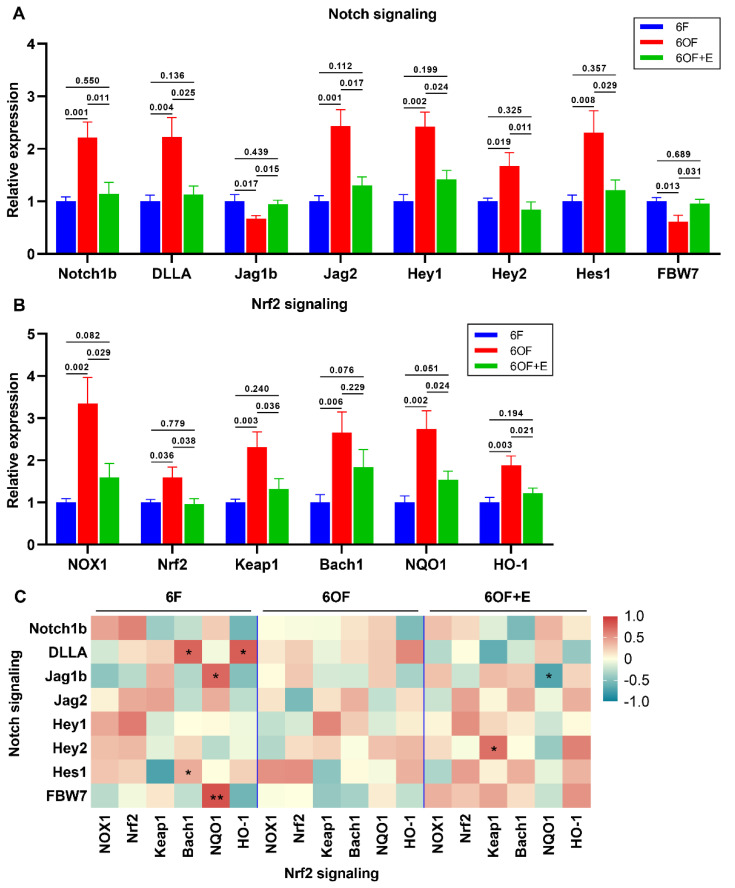
Notch-Nrf2 crosstalk was active to oxidative stress amelioration in the liver of *M. amblycephala*. (**A**,**B**), Transcriptional expression of key related genes under oxidative stress and emodin stimulation. (**A**), Notch signaling, Notch1b, Delta like A (DLLA), Jagged 1 beta (Jag1b), Jagged 2 (Jag2), Hairy/Enhancer of split related with YRPW motif 1 (Hey1), Hairy/Enhancer of split related with YRPW motif 2 (Hey2), hairy and enhancer of split-1 (Hes1), F-box and WD repeat domain-containing 7 (FBW7); (**B**), Nrf2 signaling, NADPH oxidase 1 (NOX1), Nuclear factor erythroid 2–related factor 2 (Nrf2), Kelch-like ECH-associated protein 1 (Keap1), BTB Domain and CNC Homolog 1 (Bach1), NAD(P)H quinone dehydrogenase 1 (NQO1), Heme oxygenase 1 (HO-1); (**C**), correlation analysis between Notch and Nrf2 signaling was retrieved from Pearson analysis. In (**C**), * represents *p* < 0.05, and ** represents *p* < 0.01. Data were analyzed by Students’ *t*-test, results were indicated as mean ± SEM, *n* = 9.

According to our previous study, Notch and Nrf2 signaling function importantly in the antioxidant regulation in the intestine. In the present study, we found Notch signaling (Figure 6A) and Nrf2 signaling (Figure 6B) were all positively activated under oxidative stress (6OF), while were all restored to the control level with the administration of emodin (6OF+E). With Pearson analysis, Notch and Nrf2 signaling was confirmed to be synthetically regulated under quiescent condition (6F, Figure 6C). However, the expression of key elements in Notch and Nrf2 signaling exhibited no significant correlation under oxidative stress (6OF), indicating the crosstalk was terminated (Figure 6C). Dietary supplement with emodin (6OF+E) recovered the crosstalk, evidenced by the correlation between Jag1b-NQO1 and Hey2-Keap1 (Figure 6C).

### 3.7. Hypothetical Regulation of Notch-Nrf2 Crosstalk on Oxidative Stress Amelioration

**Figure 7:**
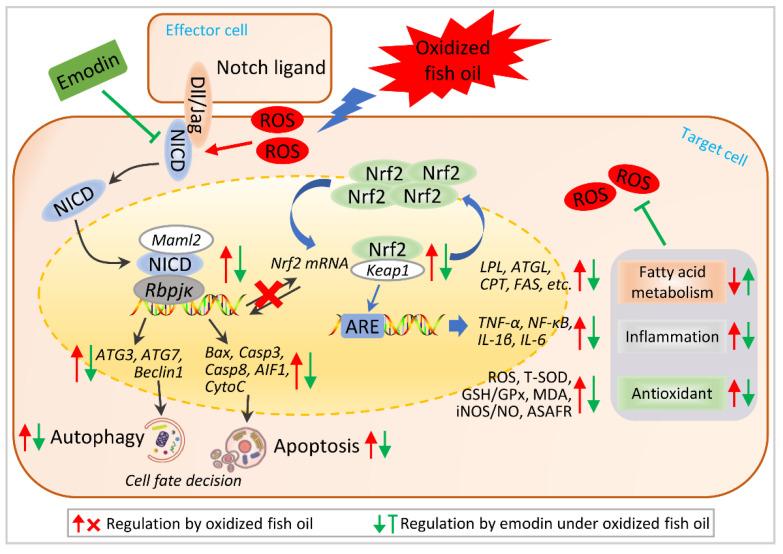
Hypothetical regulation of Notch-Nrf2 crosstalk on oxidative stress amelioration. Hypothetical regulation of Notch-Nrf2 crosstalk was raised based on the results in this study. Arrows in red color represent the regulation by oxidized fish oil, in blue color represent the regulation by emodin under oxidized fish oil. “×” represents oxidized fish oil blocks the co-regulation between Notch and Nrf2 signaling.

Based on the above results, we raise the hypothetical regulation schematic of Notch-Nrf2 crosstalk under oxidative stress amelioration (Figure 7). Oxidative stress-induced ROS activates Notch1b signaling and subsequently activates Nrf2 signaling, evidenced by the crosstalk between Notch ligands and down-stream modulators with Nrf2 key elements. Activated crosstalk of Notch-Nrf2 increased apoptosis and autophagy in the liver, which facilitates the cells to resist oxidative stress. Exhilaratingly, dietary emodin inhibited Notch-Nrf2 crosstalk to ameliorate apoptosis and autophagy, thereby alleviating oxidative stress.

## 4. Discussion

Fish oil is enriched with HUFAs that could dominantly promote the growth of fish. However, fish oil is prone to oxidative rancidity, and oxidated diet is one of the most important exogenous factors leading to oxidative stress on fish [19]. Oxidative stress control and prevention have become a critical issue for aquatic animals [8]. In aquatic animals, unavoidable oxidation of dietary lipids could induce lipid peroxidation, which increases ROS production during cellular metabolism [19]. Our previous study indicates dietary oxidized lipids-induced ROS production exceeded the ability to quench the reactive species in the intestine, thereby impaired the cellular structural integrity, suppressed the immunity and antioxidant capacity of the intestine, and eventually inhibited the growth performance of *M. amblycephala* [1,17,19]. However, as the crucial organ for metabolism, immunity and antioxidant resistance, the role and underlying mechanism of liver in resisting dietary oxidized lipids remains unclear in *M. amblycephala*.

As aquatic animals contain high amounts of lipids with polyunsaturated fatty acid residues that is the substrate for oxidation, oxidative stress could induce lipid peroxidation thereby disrupting the lipid metabolism. Generally, due to the high damaging capacity and biological activity, the cellular metabolism of ROS is under fine control in quiescent conditions. However, when steady-state ROS concentration is transiently or chronically enhanced, excessive accumulation of ROS could damage cellular constituents, and disrupt metabolic and antioxidant capacity [20]. In the present study, elevated ROS reveal dietary oxidized fish oil-induced oxidative stress in *M. amblycephala* liver. Meanwhile, as the end product of lipid peroxidation, MDA concentration was increased under 6OF, indicating dietary oxidized fish oil-induced lipid peroxidation in the liver of *M. amblycephala*.

Morphologically, cellular integrity is vital for the function of liver cells [21]. Nucleus is the control center of the cell that contains most of the genomic DNA and all of the chromosome, it maintains the integrity of genes and controls the activities of the cell by regulating gene expression [22]. Mitochondrion is the dominant organelle to produce energy through respiration that is critical for any metabolism and cellular activity [23]. Lipid droplets are lipid-rich cellular organelles to regulate the storage and hydrolysis of neutral lipids, they play a very important role in the regulation of intracellular lipid storage and lipid metabolism. Meanwhile, lipid droplets include a close association to inflammatory responses [24], lipotoxicity protection [25], as well as a platform for protein binding and degradation [26]. In the present study, TEM reveals oxidative stress-induced nucleus aggression, thereby impairing the cell fate evidenced by apoptosis and autophagy-related gene expression, indicating the metabolic disorder and cell fate determinate dysregulation in the liver.

Glucose is regarded as the major substrate for animals, and amino acids and fatty acids are also intermediates of the metabolic pathway to drive energy production [27]. Meanwhile, evidence indicate that glycolytic metabolism closely interacts with fatty acid and amino acid metabolic profiles to maintain normal cellular function, homeostasis, as well as cell fate determination [27]. It is clear that long-term administration of oxidized fish oil could induce lipid deposition in the liver [28]. In this study, oxidized fish oil led to lipid deposition in the liver, as evidenced by lipid droplets enlargement and accumulation. Similar results were also found in channel catfish [29], yellow catfish [30], loach [31], and largemouth bass [32]. In the study of channel catfish, lipid deposition in the liver induced by oxidized fish oil was originated from activated lipid synthesis [29]. However, lipid synthesis and lipolysis were both inhibited under oxidative stress in our present study, which was supported by decreased lipase, LPL, TG, TC, and FAS activity, as well as decreased lipid metabolic related gene expression of LPL, ATGL, CPT1, CPT2, COX2, FAS, and SREBP1. This contrasting result reveals there might have different regulatory mechanisms among different fishes under oxidative stress, or the regulatory mechanism was specific to different oxidative stress levels. Therefore, we suppose the oxidative stress was too severe to maintain lipid synthesis and lipolysis, thereby results in lipid deposition and inhibited lipid metabolism in our study. However, the underlying mechanism needs further investigation. Additionally, glycolytic and amino acid metabolism were also inhibited under oxidized fish oil and restored by emodin, indicating that nutrient metabolisms are closely associated with each other, and the regulation of the metabolic pathway could be important in oxidative stress resistance.

Cholesterol is a sterol type of lipid that serves as an essential structural component of animal cell membranes and a precursor for the biosynthesis of steroid hormones, bile acid, and vitamin D. Early research identifies cholesterol itself as an antioxidant that protects cells from free radical damage [33]. Interestingly, cholesterol was significantly reduced under oxidative stress in our present study. Mechanically, cholesterol was mainly synthesized from dietary lipid with fatty acid metabolism [34]. From this point of view, if the dietary lipid was oxidized to induce peroxidation for the organism, the synthesis of cholesterol will be unavoidably dysregulated. Consistently, reports indicate that oxidative damage could deplete cholesterol and disrupt cell membrane function [35,36]. Meanwhile, peroxidation-induced cellular structural impairment might be another factor to reduce cholesterol level in the liver. Additionally, it is clear that emodin could reduce cholesterol level [37,38]. In our study, cholesterol in 6OF+E was reduced in comparison with 6F, indicating ameliorative effects of emodin on oxidative stress.

Impaired lipid metabolism was reported to interact with immunity and antioxidant dysregulation [39]. iNOS-derived NO signaling plays a central role in inflammatory regulation and hepatic protection [40]. Meanwhile, interferon system was reported to induce iNOS via activating NF-κB [41]. In the present study, NF-κB, IL-1β, and IL-6 expression was activated and iNOS content was increased under oxidized fish oil stimulation, indicating the immune system was activated, which was in accordance with that in zebrafish [42]. Antioxidant system functions importantly to remove the excessive ROS and ASAFR formation, and SOD and GPx are vital to decrease hydrogen peroxide in this process [43,44]. The present study shows that oxidized fish oil markedly increased T-SOD, GPx, and ASAFR activity, as well as ROS and MDA levels, indicating antioxidant system was activated in the liver of *M. amblycephala*. Similar studies also confirmed that oxidized fish oil dysregulates antioxidant capacity in channel fish [29] and tilapia [45].

Recent advances in physiological relevance reveal ROS acts as an important signaling molecule as well as a critical factor in cell fate determination [27]. Previous evidence suggests that ROS acts as signaling mediators linking between metabolic alteration and cell fate, such as cell cycle progression, apoptosis, and autophagy [46]. In this study, autophagy and apoptosis-related genes were all activated under oxidized fish oil stimulation, indicating prolonged oxidative stress results in apoptosis and autophagy.

Our previous reports reveal cell fate determination-related apoptosis and autophagy were closely related to Nrf2 and Notch signaling [1,17], which inspired us to uncover the relationship between apoptosis-autophagy and Nrf2-Notch signaling. Activation of Nrf2-ARE pathway protects cells from oxidative stress-induced cell death [47]. Notch signaling plays an important role in the process of cell fate determination, including cell growth, cell proliferation, and programmed death [48]. Through Notch-Nrf2 crosstalk studies, it has been clearly shown that NICD can activate the Nrf2 pathway and Nrf2 can inversely activate the Notch1 pathway in the liver [11]. Recent studies also indicate ARE is the upstream of the Notch1 major transcription start site. Furthermore, as the binding site of NICD, Rbpjκ is conserved on the promoters of Nrf2 among animal species [49]. Notch1 is one of the transmembrane Notch family receptors that drive Notch signaling, together with the Rbpjκ transcription factor [50]. Therefore, the crosstalk between Nrf2 and Notch signaling has been shown to enhance cyto-protection and maintenance of cellular homeostasis under oxidative stress, rather than as a simple on-off switch [51]. In our present study, oxidized fish oil robustly activated Nrf2 and Notch signaling, and induced aberrant Nrf2-Notch crosstalk by molecular impairment of related key genes. Under emodin stimulation, Nrf2 and Notch signaling was ameliorated and the crosstalk was restored, indicating the inhibition of Nrf2 and Notch signaling, as well as the promotion of Nrf2-Notch crosstalk plays a key role in liver development and in maintenance of hepatic function under oxidative stress. In accordance with our finding, Notch1-Nrf2 crosstalk exerts cellular protection by reducing the formation of ROS [52], promoting apoptosis and aggravating tight joint or oxidative damage [53]. Additionally, Notch or Nrf2 inhibition has been well used as a therapeutic target in the treatment of oxidative stress with different bioactive compounds, such as emodin [17], quercetin [54], and chlorogenic acid isomers [55] on Nrf2; as well as emodin [1], dibenzazepine [12], melatonin [56], and niclosamide [57] on Notch. We speculate that the ROS-Notch-Nrf2 pathway is a conserved pathway that is designed to allow cells to respond to changes in environmental levels of ROS. However, there are limited reports illustrating the breakdown of Nrf2-Notch crosstalk under oxidative stress. Presumably, a system for downregulation of this crosstalk should exist for the homeostasis maintenance, perhaps target gene products of each signaling contribute to a negative feedback mechanism for Notch and/or Nrf2 signaling regulation; the detailed mechanism requires further investigation. Our findings reveal, to our knowledge, the first observation of ROS activation and Notch-Nrf2 signaling in response to oxidative stress amelioration of *M. amblycephala*. Additionally, our study is supported by the previous report of Nrf2-Notch interaction in total liver cells [58].

To combat the adverse effects of external stress, especially the oxidative stress that could induce cellular lipid peroxidation, homeostasis imbalance, immune or antioxidant breakdown and even death, dietary implementation with functional additives has become an effective approach for stress control and prevention in aquatic animals [59]. For the characterized advantages of minor side effects and drug resistance, the medical herb extract emodin has been widely used in clinical trial for humans and aquaculture [59]. In consistent with our previous results that emodin protects the intestine from oxidative stress impairment [1,17,18], we demonstrate emodin alleviates morphological impairment, fatty acid metabolic disorder, antioxidant disorder, and cell fate determination by targeting Notch-Nrf2 crosstalk in the liver of *M. amblycephala*. Similar functional effects of emodin was also reported in other animals [60,61,62,63].

Taken together, our study emphasizes that the loss of normal ROS-Notch-Nrf2 cellular homeostatic mechanism was associated with excessive autophagy, apoptosis, and metabolic disorder in the liver. Meanwhile, our data demonstrate a vital homeostatic mechanism that emodin prevents excessive stress injury of liver cells and allows them to respond to injury and cellular repair.

## 5. Conclusions

In conclusion, dietary oxidized lipid induced oxidative stress and dysregulated lipid metabolism in the liver of *M. amblycephala.* Singular activation of Nrf2 and Notch signaling, interrupted Nrf2/Notch1 crosstalk, as well as apoptosis and autophagy were involved in the regulation. Furthermore, the therapeutic Nrf2-Notch crosstalk targeting holds great promise for the treatment of oxidative stress in aquatic animals, which could promote the application of emodin from scientific research into aquaculture practice.

## Figures and Tables

**Table 1:** Formulation and proximate composition of experimental diets.

Ingredient/%	6F	6OF	6OF+E	Nutrition Value (%, Dry Matter)	6F	6OF	6OF+E
Casein	25.0	25.0	25.0	Dry matter, DM	92.06	92.18	92.24
Gelatin	5.0	5.0	5.0	Crude protein, CP	33.11	33.11	33.11
Fish meal	10.0	10.0	10.0	Crude lipid	7.01	7.01	7.01
Dextrin	10.0	10.0	10.0	Nitrogen Free Extract, NFE	1.00	1.06	1.09
α-starch	24.5	24.5	24.5	Ash	8.28	8.34	8.37
Fish oil	6.0	0.0	0.0	Ca	1.62	1.62	1.62
Oxidized fish oil	0.0	6.0	6.0	Total P	1.05	1.05	1.05
Microcrystalline cellulose	7.0	7.0	7.0	Lysine	2.49	2.49	2.49
Carboxymethylcellulose	5.0	5.0	5.0	Cysteine	0.92	0.92	0.92
Choline chloride	1.0	1.0	1.0	Methionine	0.19	0.19	0.19
Vitamin premix ^a^	1.0	1.0	1.0	Threonine	1.34	1.34	1.34
Mineral premix ^b^	1.0	1.0	1.0	Arginine	1.50	1.50	1.50
Calcium dihydrogen phosphate	2.0	2.0	2.0	Fe	33.70	33.70	33.70
Attapulgite	2.0	2.0	2.0	Gross Energy ^c^	14.68	14.68	14.68
Ethoxyquin	0.5	0.5	0.5				
Total	100.0	100.0	100.0				
Emodin (mg/kg)	0.0	0.0	30.0				

Note: ^a^ Vitamin contents per kg diets: Vitamin A, 9000 IU; Vitamin B_1_, 3.2 mg; Vitamin B_2_, 10.9 mg; Vitamin B_5_, 20 mg; Vitamin B_6_, 5 mg; Vitamin B_12_, 0.016 mg; Vitamin C, 50 mg; Vitamin D, 2000 IU; Vitamin E, 45 mg; Vitamin K_3_, 2.2 mg; Niacin, 28 mg; Folic acid, 1.65 mg; Pantothenate, 10 mg; Choline, 600 mg. ^b^ Mineral contents per kg diets: FeSO_4_·7H_2_O, 250 mg; CuSO_4_·5H_2_O, 20 mg; ZnSO_4_·7H_2_O, 220 mg; Na_2_SeO_3_, 0.4 mg; MnSO_4_·4H_2_O, 70 mg; CoCl_2_·6H_2_O, 1 mg; KI, 0.26 mg; ^c^ Energy, calculated by using standard physiological fuel values of 37.7, 16.7, and 16.7 kJ g^−1^ for protein, lipid and carbohydrate, respectively.

**Table 2:** Primers and sequences referred in the experiment.

Gene	Primer	Sequence (5′→3′)	Accession No.	Gene	Primer	Sequence (5′→3′)	Accession No.
*LPL*	F	TTACAGGCTGAGATTGACTA	KF114279.1	*AIF1*	F	GGATTTTCCTCGCACAAAAC	XM_048210652.1
	R	GAAGAACATCCACGAAAA			R	TGTCGTCTGTGGCTTCACTT	
*ATGL*	F	ATCCTTGTATCCCTGCTTG	KX010807.1	*CytoC*	F	GCACAAAGTCGGTCCAAATC	XM_048185968.1
	R	GTGACAGACGGAGAAAACG			R	GCTCTCTCGCCCTTCTTCTT	
*CPT1*	F	TACTTCCAAAGCGGTGAG	KJ141198.1	*Notch1b*	F	GCGATTATGGAAGGTGCATT	XM_048189338.1
	R	AGAGGTATTGTCCGAGCC			R	GTCGTGATACCCCTCTCTGC	
*CPT2*	F	CCATAGCCCACTCCGAAAC	XM_048208951.1	*DLLA*	F	TGACAACAGAAAACCCAGAGC	XM_048205545.1
	R	TGCCGCCATAAACCACAA			R	TCCCTCGCCATAGTAGTGCT	
*Cox2*	F	AACCCAGGACCTTACACCC	NC_010341.1	*Jag1b*	F	GTAAACGGAGGGCAGTGTGT	XM_048205450.1
	R	CCCGCAGATTTCAGAACA			R	GCGCACTTGTAGCTTCCTTC	
*UCP2*	F	TGGCTACAGCACAGTTGAGG	XM_048179976.1	*Jag2*	F	CTTCCTGACGTGCCTCTCTC	XM_048205375.1
	R	TGACCTCATCAAAGATGCAC			R	GTGGGCAGTTTGTCCTTGTT	
*FAS*	F	AGCGAGTACGGTGATGGT	KF918747.1	*Hey1*	F	GGGCTCACACCACCTACAAC	XM_048201329.1
	R	GGATGATGCCTGAGATGG			R	CCCTATTTCCATGCTCCAAG	
*SREBP1*	F	ACAACAGTAGCGACACCCTG	MH633449.1	*Hey2*	F	AACGGCATTTGAGAAACAGG	XM_048207495.1
	R	AGGAGCGGTAGCGTTTTTCA			R	GCTGAGGTGAGAAACCAAGC	
*IL-1β*	F	CGATAAGACCAGCACGACCTT	MN294974.1	*Hes1*	F	CCTGCTTTCGCTTCTGCTAC	XM_048186605.1
	R	GTTTCCGTCTCTCAGCGTCA			R	GCACTAACACCAACGGGACT	
*IL-6*	F	GTCCTCTGCCGGTCAAATC	KJ755058.1	*FBW7*	F	CTGAAACCGAGACCTGCCTA	XM_048197725.1
	R	CAGTCGCTGGGTCTCTTCAC			R	CTGATGACCTGTGAGCGTGT	
*TNF-α*	F	CTGTCTGCTTCACGCTCAAC	KU976426.1	*NOX1*	F	CTGGCTGCTCATCACAGAAG	XM_048176512.1
	R	GGTCCTGGTTCACTCTCCAA			R	CCACTATCGCTGGTCTCACA	
*NF-κB*	F	GGGTTTTTCATTGGTGGATG	MK315050.1	*Nrf2*	F	AAGAGCGAACGTAGCACCAG	XM_048178958.1
	R	GCAGAACTGTGGCAATCTGA			R	GCAGTGTGCTGAAGGGAGTAT	
*ATG3*	F	CGCCAGTTTTGAAGGAATCT	XM_048200321.1	*Keap1*	F	GAGATTCGCAGAGGAGATCGG	XM_048162397.1
	R	TTGTCTTTGGGCAGATAGGG			R	CTGGCAATGGGACAAGCTGA	
*ATG7*	F	ATCACACCAGGAGCGTCTTT	XM_048172227.1	*Bach1*	F	CAGCCATCATTTCCAACCTT	XM_048160868.1
	R	GGTTCATTCATCCGGTCATC			R	GAGACGCCTGACAAGAATCC	
*Beclin1*	F	TCGACACATCCTTCAACGTC	XM_048187618.1	*NQO1*	F	AAGCCTCTGTCCTTTGCTCC	XM_048186312.1
	R	ATGTATTTCCGAGCCACACC			R	TCTGGAGGAAGTGGTTTGCC	
*Bax*	F	CCCCCTCATCTTTCCATTCT	MK315043.1	*HO-1*	F	CAGGAGCAGAATGAACAGCA	KU382526.1
	R	CAAACATCCCCTTTCTTCTCC			R	CCAAAGTGATTCCCACACCT	
*Casp3*	F	AGATGGTGTGGGAGATGGAG	KY006115.1	*β-actin*	F	TCTGCTATGTGGCTCTTGACTTCG	AY170122.2
	R	CCAGTTGCTTGCCGTATTTT			R	CCTCTGGGCACCTGAACCTCT	
*Casp8*	F	TTGTCTGCTGTGTCCTCTCG	XM_048200589.1				
	R	ATCGTTCCCTTGTCCATCTG					

Note: The mRNA sequences for each gene were obtained from *M. amblycephala* transcriptome sequencing database. Primers for RT-PCR were designed using primer premier 5.0.

**Table 3:** Fatty acid composition in the diet and *M. amblycephala*.

	Feed			*M. amblycephala*		
	6F	6OF	6OF+E	6F	6OF	6OF+E
C12:0	0.071 ± 0.006 b	0.106 ± 0.003 a	0.120 ± 0.002 c	0.044 ± 0.002 A	0.044 ± 0.002 A	0.054 ± 0.002 A
C14:0	1.061 ± 0.035 b	2.549 ± 0.028 a	2.596 ± 0.113 a	1.584 ± 0.049 A	1.554 ± 0.031 A	1.688 ± 0.051 A
C15:0	0.132 ± 0.018 b	0.583 ± 0.139 a	0.453 ± 0.002 a	0.224 ± 0.002 A	0.235 ± 0.023 A	0.253 ± 0.031 A
C16:0	14.539 ± 0.889 b	33.132 ± 0.521 a	32.591 ± 0.341 a	21.789 ± 0.456 A	20.960 ± 0.554 A	22.351 ± 0.779 A
C17:0	0.231 ± 0.018 c	1.236 ± 0.021 a	1.099 ± 0.011 b	0.355 ± 0.020 AB	0.331 ± 0.018 B	0.437 ± 0.026 A
C18:0	4.425 ± 0.245 b	6.296 ± 0.171 a	6.072 ± 0.042 a	7.263 ± 0.152 A	6.626 ± 0.073 A	6.666 ± 0.385 A
C20:0	0.343 ± 0.025 a	0.211 ± 0.006 b	0.249 ± 0.011 b	0.103 ± 0.002 A	0.113 ± 0.008 A	0.126 ± 0.015 A
**ΣSFA**	**20.802 ± 1.237 b**	**44.113 ± 0.874 a**	**43.180 ± 0.521 a**	**31.362 ± 0.683 A**	**29.863 ± 0.708 A**	**31.575 ± 1.288 A**
C16:1	1.959 ± 0.034 b	12.239 ± 0.081 a	13.258 ± 0.150 a	6.604 ± 0.233 B	8.409 ± 0.121 A	8.187 ± 0.108 A
C18:1	22.940 ± 1.120 b	27.270 ± 0.156 a	26.029 ± 0.595 a	33.519 ± 0.878 B	39.298 ± 0.751 A	35.979 ± 1.721 AB
C20:1	0.311 ± 0.006 b	0.681 ± 0.047 a	0.624 ± 0.072 a	0.758 ± 0.010 B	0.960 ± 0.035 A	0.806 ± 0.061 AB
C22:1	0.150 ± 0.006 c	0.247 ± 0.010 a	0.203 ± 0.002 b	0.049 ± 0.005 A	0.022 ± 0.004 B	0.063 ± 0.008 A
**ΣMUFA**	**25.360 ± 1.166 b**	**40.437 ± 0.293 a**	**40.115 ± 0.818 a**	**40.930 ± 1.126 B**	**48.689 ± 0.910 A**	**45.035 ± 1.897 AB**
C18:2	10.689 ± 9.657 a	5.084 ± 0.049 b	4.978 ± 0.103 b	5.789 ± 0.051 A	4.102 ± 0.059 B	3.683 ± 0.221 B
C18:3n6	0.672 ± 0.007 a	0.390 ± 0.017 b	0.476 ± 0.015 b	0.151 ± 0.001 A	0.133 ± 0.019 A	0.141 ± 0.012 A
C18:3n3	5.167 ± 0.096 a	1.187 ± 0.062 c	1.606 ± 0.073 b	0.628 ± 0.010 A	0.408 ± 0.005 B	0.443 ± 0.025 B
C20:2	0.223 ± 0.179 a	0.063 ± 0.002 b	0.046 ± 0.003 b	0.153 ± 0.002 C	0.877 ± 0.044 A	0.548 ± 0.048 B
C20:3	0.241 ± 0.001 a	0.139 ± 0.005 b	0.131 ± 0.006 b	0.786 ± 0.015 A	0.750 ± 0.029 AB	0.658 ± 0.033 B
C20:4	1.182 ± 0.018 a	0.664 ± 0.008 b	0.754 ± 0.002 b	1.701 ± 0.058 A	1.732 ± 0.018 A	1.627 ± 0.073 A
C20:5	12.205 ± 0.061 a	5.665 ± 0.094 b	6.685 ± 0.043 b	4.776 ± 0.044 A	2.450 ± 0.202 C	3.455 ± 0.263 B
C22:3	0.118 ± 0.006 a	0.034 ± 0.002 b	0.022 ± 0.001 b	0.103 ± 0.002 A	0.147 ± 0.021 A	0.126 ± 0.015 A
C22:4	0.246 ± 0.003 a	0.090 ± 0.006 b	0.060 ± 0.006 b	0.269 ± 0.011 A	0.389 ± 0.051 A	0.350 ± 0.017 A
C22:5	0.840 ± 0.012 a	0.309 ± 0.005 b	0.247 ± 0.004 b	1.910 ± 0.064 A	1.095 ± 0.055 B	1.400 ± 0.115 B
C22:6	12.437 ± 0.021 a	1.917 ± 0.068 b	1.686 ± 0.021 b	11.449 ± 0.260 A	9.379 ± 0.161 B	10.929 ± 0.537 A
**ΣPUFA**	**44.020 ± 9.285 a**	**15.542 ± 0.316 b**	**16.692 ± 0.267 b**	**27.715 ± 0.517 A**	**21.462 ± 0.665 B**	**23.177 ± 1.174 B**

Note: letters “a, b, and c” represent the difference in the feed, “A, B, and C” represent the difference in the *M. amblycephala*.

## Data Availability

Data is contained within the article.

## Supplementary Materials

The following supporting information can be downloaded at: https://www.mdpi.com/article/10.3390/antiox11061179/s1, Figure S1: Interaction analysis between fatty acid metabolism and antioxidant capacity; Figure S2: Interaction analysis among inflammation, apoptosis, and autophagy.
