# Supplementary material for: Protective Effects of Emodin on Oxidized Fish Oil-Induced Metabolic Disorder and Oxidative Stress through Notch-Nrf2 Crosstalk in the Liver of Teleost Megalobrama amblycephala
Source: Antioxidants (Basel). 2022 Jun 15;11(6):1179. doi: 10.3390/antiox11061179 (PMC9219933; doi:10.3390/antiox11061179)
Supplement: Supplementary file 1 [file antioxidants-11-01179-s001.zip › antioxidants-1743490-supplementary.pdf]

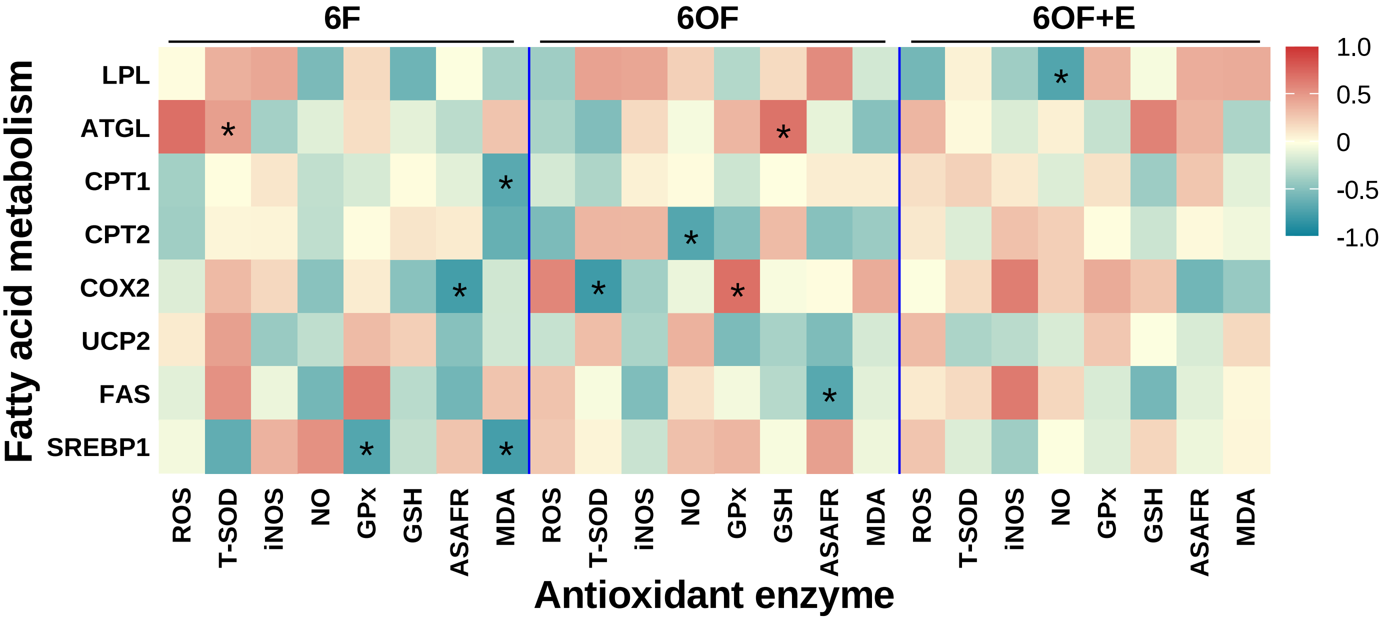

Figure S1. Interaction analysis between fatty acid metabolism and antioxidant capacity

Correlation analysis between fatty acid metabolism and antioxidant enzyme related parameters were retrieved from Pearson analysis. Data were analyzed by Students' *t*-test; * represents $p < 0.05$, and ** represents $p < 0.01$; results were indicated as mean ± SEM, n=9.

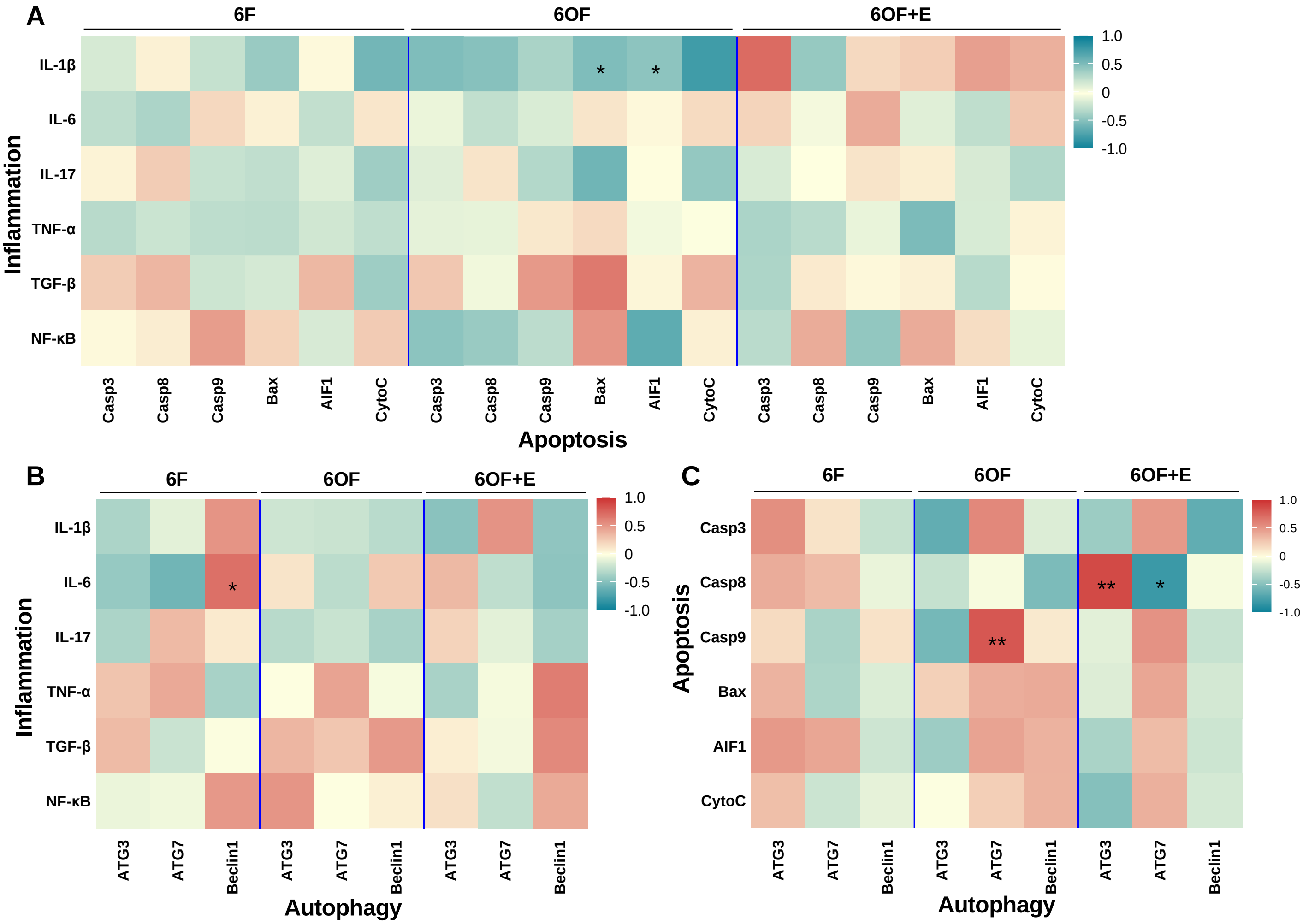

Figure S2. Interaction analysis among inflammation, apoptosis, and autophagy

Correlation analysis was retrieved from Pearson analysis. A) correlation between inflammation and apoptosis related key genes; B) correlation between inflammation and autophagy; C) correlation between apoptosis and autophagy. Data were analyzed by Students' *t*-test; * represents $p < 0.05$, and ** represents $p < 0.01$; results were indicated as mean ± SEM, n=9.
